# Global mapping of binding sites for phic31 integrase in transgenic maden-darby bovine kidney cells using ChIP-seq

**DOI:** 10.1186/s41065-018-0079-z

**Published:** 2019-01-14

**Authors:** Lijuan Qu, Lei Wang, Xueyuan Zhu, Yan Zhang, Qiang Ou, Aying Ma, Fengying Sheng, Xiaoqing Wei, Yue Dai, Guoting Li, Shuwu Xie

**Affiliations:** 1 Department of Laboratory Medicine, Shanghai Eighth People’s Hospital, Shanghai, 200040 China; 2 Department of Respiratory Medicine, Shanghai First People’s Hospital, Shanghai Jiaotong University School of Medicine, Shanghai, 201620 China; 3 Lab of Reproductive Pharmacology, NHC Key Lab of Reproduction Regulation, Shanghai Institute of Planned Parenthood Research, Fudan University, Shanghai, 200032 China

**Keywords:** ΦC31 integrase, Binding sites, ChIP-seq, Sequence motif

## Abstract

**Background:**

ΦC31 integrase, a site-specific recombinase, can efficiently target attB-bearing transgenes to endogenous pseudo attP sites within mammalian genomes. The sequence features of endogenous binding sites will help us to fully understand the site-specific recognition function by ΦC31 integrase. The present study was aimed to uncover the global map of ΦC31 integrase binding sites in bovine cells and analysis the features of these binding sites by comprehensive bioinformatics methods.

**Results:**

In this study, we constructed a ChIP-seq method that can be used to uncover the global binding sites by phiC31 integrase. 6740 potential ΦC31 integrase binding sites were identified. A sequence motif was found that contains inverted repeats and has similarities to wild-type attP site. Using REPEATMASKER, we identified a total of 20,183 repeat-regions distributed in 50 repeat types for the 6740 binding sites. These sites enriched in “regulation of GTPase activity” of in the GO category of biological process and KEGG pathway of signal transmembrane transporter activity.

**Conclusion:**

This study is the first time to uncover the global map of binding sites for ΦC31 integrase using ChIP-sequencing method and analysis the features of these binding sites. This method will help us to fully understand the mechanism of the site-specific integration function by phiC31 integrase and will potentially boost its genetic manipulations in both gene therapy and generation of transgenic animals.

**Electronic supplementary material:**

The online version of this article (10.1186/s41065-018-0079-z) contains supplementary material, which is available to authorized users.

## Background

The Streptomyces phage ΦC31 integrase is a site-specific recombinase that catalyzes recombination between two short DNA sequences, a bacterial attachment site (attB) and a phage attachment site (attP) [1]. As prokaryotic attachment sites, attP and attB are unlikely to occur in the genomes of mammals and most plants, but pseudo attP sites are present in the eukaryotic genome [2–5], ΦC31 integrase can efficiently target attB-bearing transgenes to endogenous pseudo attP sites within mammalian genomes [6, 7]. Because ΦC31 integrase-mediated recombination is stable, irreversible and does not require external chemical energy and cofactors, ΦC31 integrase has become a powerful tool for both gene therapy animals [8–13] and generation of transgenic animals [14–16]. However, with the use of ΦC31 integrase system in genetic manipulations, an increasing number of problems is being encountered. On one hand, the presence of a large number of pseudo attP sites in the genome increases the risk of chromosomal rearrangements and DNA damage [17]. On the other hand, due to the high activity of the integrase system in the genome, the specificity does not appear to be as high as expected. Therefore, it is necessary to further study the mechanism of ΦC31 integrase system in mammalian cells, in order to further optimize the system, improve its integration specificity and reduce the non-specific recombination.

Studies on sequence characteristics of pseudo attP sites in many species revealed that sequence characteristic is one of the most important factors for ΦC31 integrase to recognize the pseudo attP sites and mediate site-specific integration in eukaryotic cells [18–21]. Meanwhile the pseudo attP sites in different cell types of the same species are cell-specific. For example, the BpsM1 site we found in bovine maden-darby bovine kidney (MDBK)cell line was not detected in bovine ear fibroblasts [18]. The 19q13.31 site in the human genome is detectable in all cell types, and 3q26.31 appears only in HepG2 cells [21]. These phenomena indicate that, in addition to sequence features, the recognition and binding of the integrase to the sites are also affected by other factors such as chromatin structure. However, this is still not fully studied and understood. The research on ΦC31 integrase function in mammalian cells is still in its infancy. Indepth studies will accelerate its application in biological engineering. Especially, the main problems that need to be investigated include several aspects. First of all, although about 10^2^–10^3^ pseudo attP sites have been predicted in mammalian cells [7], the actual number of potential sites we didn’t know. Thus the data on ΦC31 integrase is too small to analyze its function and mechanism. Secondly, at present, common methods for identification of integration sites are plasmid rescue, reverse nested PCR and other methods. These methods have limitations and provide only a small amount of binding sites information.

Chromatin immunoprecipitation assay (ChIP) is a powerful tool for studying the interaction between protein and DNA in vivo. The ChIP-Seq technology, which combines ChIP with second-generation sequencing technology, can efficiently detect genome-wide DNA segments that interact with functional proteins [22].

In this study we establish an expression platform for ΦC31 integrase in bovine cells and identify the whole set of ΦC31 integrase binding sites in these cells by using ChIP-Seq technology. We equally aimed to analyze the characteristics of these binding sites using bioinformatics.

## Results

### Generation of MDKB cells expressing ΦC31 integrase

**Fig. 1:**
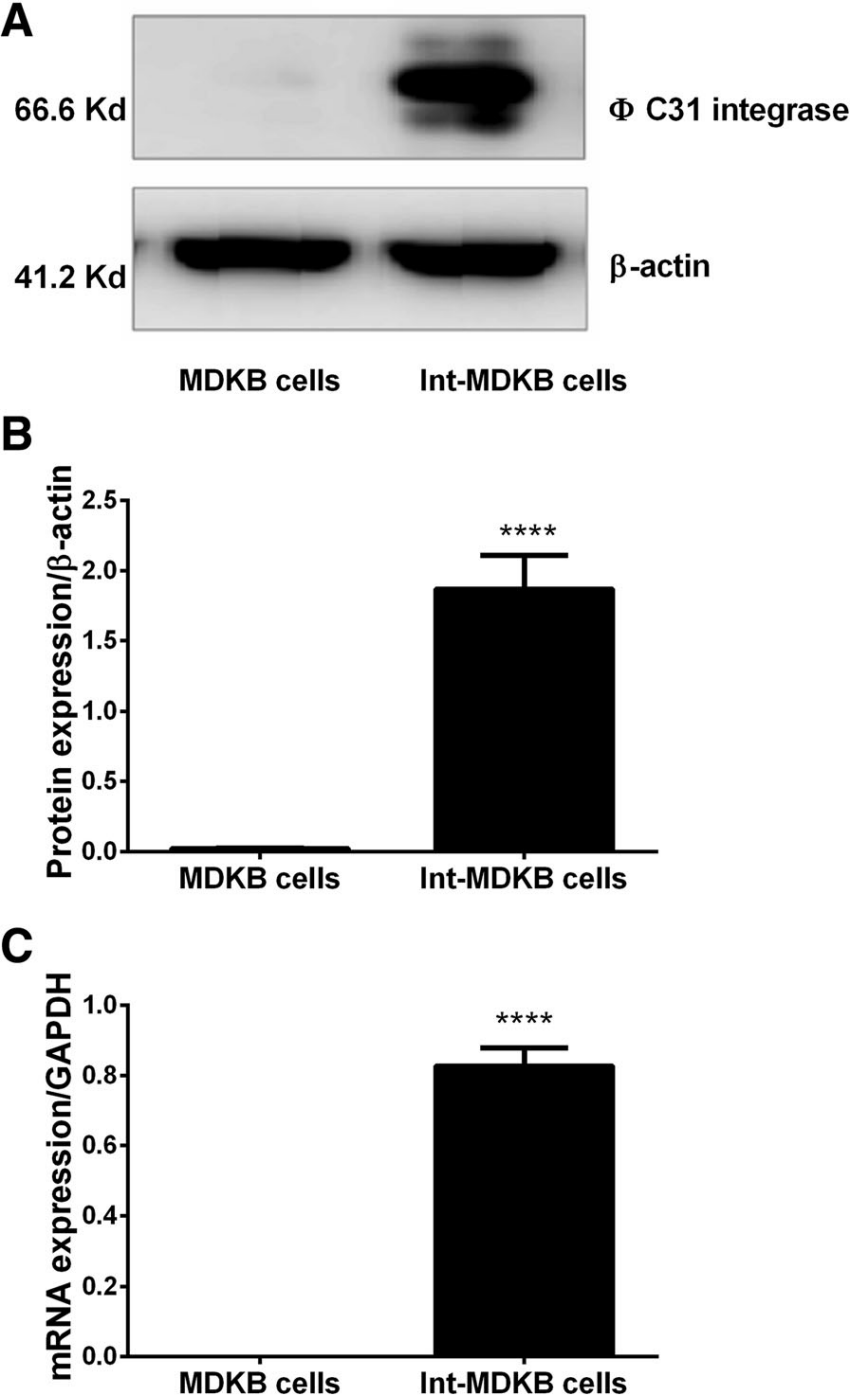
Verification of ϕC31 integraseexpression in the established MDBKcells. **a** Western blot analysis of ϕC31 integrase expression in the MDBK cells. **b** densitometric analysis of the representative bands obtained from thewestern blot analysis. **c** Real time PCR analysis of ϕC31 integrase expressionin the MDBK cells. β-actin was used as endogenous control for western blotanalysis while the GAPDHgene was employed as housekeeper gene in thereal time PCR analysis

In this study, the ΦC31 integrase gene sequence was isolated from pCMV-INT plasmid and inserted into the pcDNA3.1-neo plasmid in order to construct a eukaryotic selection vector for generating ΦC31 integrase cell lines. The constructed expression vector pcDNA3.1-int-neo (7272 bp) was validated by enzyme digestion and sequencing. MDKB cells were transfected with the constructed plasmid pcDNA3.1-int-neo. After G418 selection, PCR analysis using specific primers for ΦC31 integrase gene and only clones harboring the ΦC31 integrase gene were used for further analysis. The presence of ΦC31 integrase gene was further confirmed by sequencing. To verify the effective expression of ΦC31 integrase, western blot was performed for ΦC31 integrase in wild type and engineered MDKB cells. As shown in Fig. 1, ΦC31 integrase was expressed, indicating that a MDKB cell line expressing the ΦC31 integrase was effectively established.

### Global mapping of binding sites for ΦC31 integrase

We performed the ChIP-Seq experiments in order to uncover the whole genome binding sites for ΦC31 integrase. The depth of sequencing data of the genome was obtained by analyzing the coverage of the reference sequence and the depth information of the genome while the gene profiling and the depth distribution of the upstream and downstream intervals were obtained by analyzing the depth distribution of the gene in the interval between the gene ontology and the upstream and downstream 2 k intervals (Additional file 1: Figures S1-S4). The statistics of the ChIP-Seq data before and after quality filtering is summarized in Table 1. A total of 42,233,088 and 36,809,270 clean short reads were generated from the raw ChIP-Seq data.

**Table 1:** Statistics of the ChIP-Seq data before and after quality filtering

	Sample	Number of sequences	Base number (bp)	Q20 (%)	Q30 (%)
Raw reads	INTMDBK_input	46106506	6915975900	96.19	92.37
	INTMDBK_treat	40239504	6035925600	96.02	91.84
Clean reads	INTMDBK_input	42233088	6127524993	98.34	95.56
	INTMDBK_treat	36809270	5411758088	98.17	95.08

Following quality control, we mapped the reads to the Reference Genome Consortium database. From the 42,233,088 reads obtained from INTMDBK_input 38,407,502 were mapped to the Reference Genome Consortium database (mapping rate of 90.94%) whereas 30,675,258 were mapped from the 36,809,270 clean reads generated from the INTMDBK_treat sample (mapping rate of83.34%). These reads with unique alignment to a unique position on the genome was used for subsequent information analysis. The 66 captured sites (markers) information and location was reported in Additional file 2.

Based on a certain analysis model in the entire genome range, the peaks (ChIP-Seq enrichment regions) were scanned to obtain the data on peak location in the genome and the peak region sequence information. The peak search output file was saved into wiggle file format and uploaded to the UCSC Genome Browser for visualization. We identified a total of 6740 ChIP-Seq peaks with total peak length of 4,136,812 and peak mean length of 613.77. The total peak depth was 2,035,208 while the peak mean depth was 301.96 for the INTMDBK_treat sample.

### Features of the binding sites

**Fig. 2:**
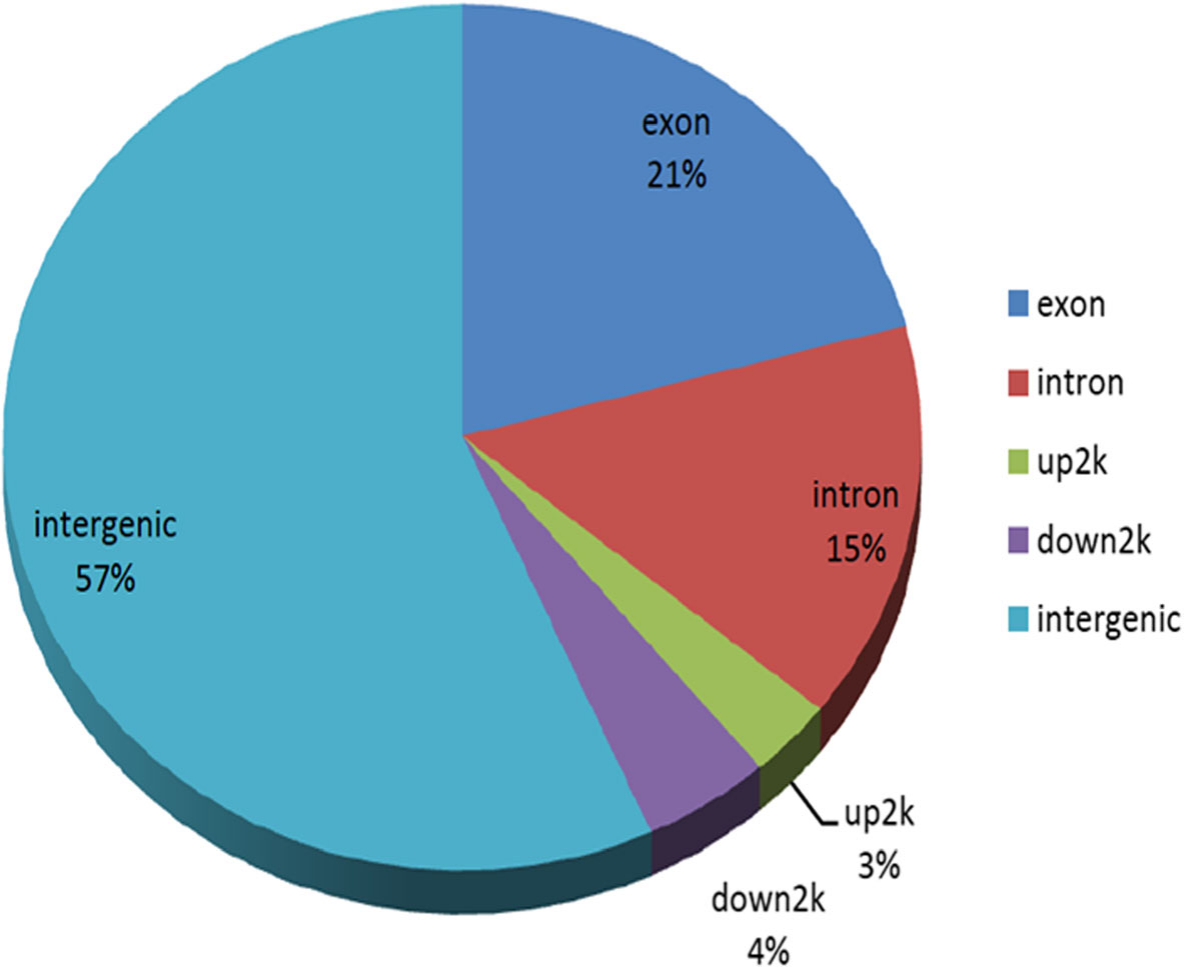
The distribution characteristics of gene functional elements of peaks. Peaks were distributed into exons, introns, upstream, downstream, intergenic and other functional elements

The distribution characteristics of gene functional elements of peaks showed that peaks were composed of exons, introns, upstream, downstream, intergenic and other functional elements (Fig. 2). The peak calling output file and peak annotation are reported in Additional file 3.

**Fig. 3:**
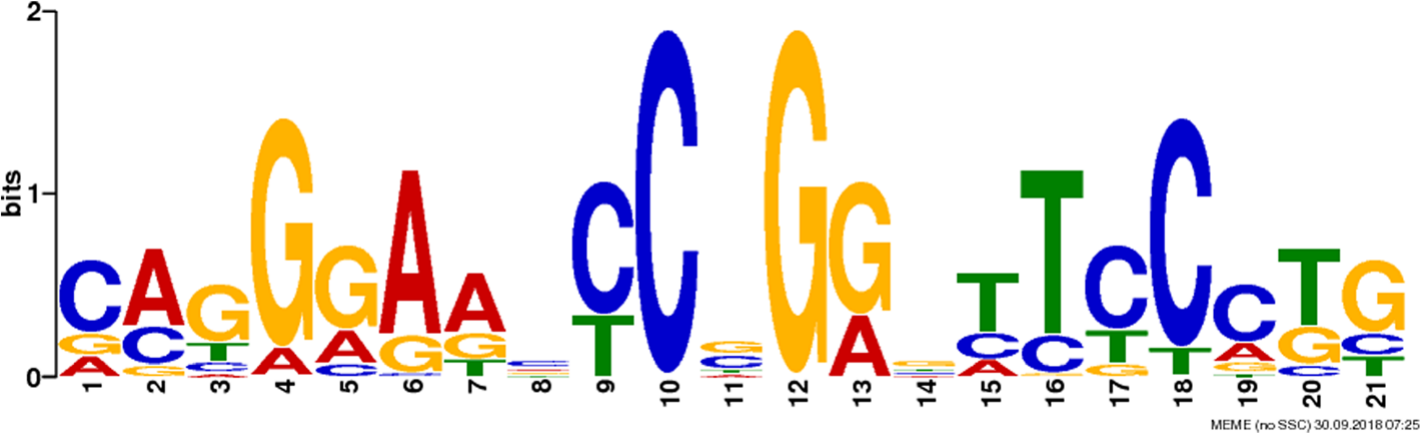
ChIP-seq peaks contain ϕC31 integrase-binding motifs. The multilevel consensus derived from the motif predicted by the MEME finder. The consensus is symmetric around the core and contains inverted repeats that extend over the length of the consensus

To determine whether our ChIP-seq experiment recovered the DNA motif assumed to be responsible for sequence-specific ΦC31 integrase–DNA binding, we applied de novo motif discovery to the ChIP-seq peak regions. Using the MEME motif discovery tool, we searched all regions for enriched sequence patterns without any assumption. A highly significant motif (E = 1.4e-043) was found in 236 sequences (Fig. 3). The motif was 21 bp long and contained inverted repeats around the core.

Using REPEATMASKER, we identified a total of 21,065 repeat-regions distributed in 50 repeat types for the 6740 binding sites (Additional file 4). The repeat-types included SINE/tRNA-Core-RTE, SINE/MIR, LINE/L1, LINE/L2, SINE/tRNA and DNA/hAT-Ac. We found that 6740 repeat-regions were located in the upstream regions of ΦC31 integrase binding sites, 6740 in the downstream and 7585 were overlapped with the binding sites.

### Molecular pathways of ChIP-Seq-based ΦC31 binding sites

**Fig. 4:**
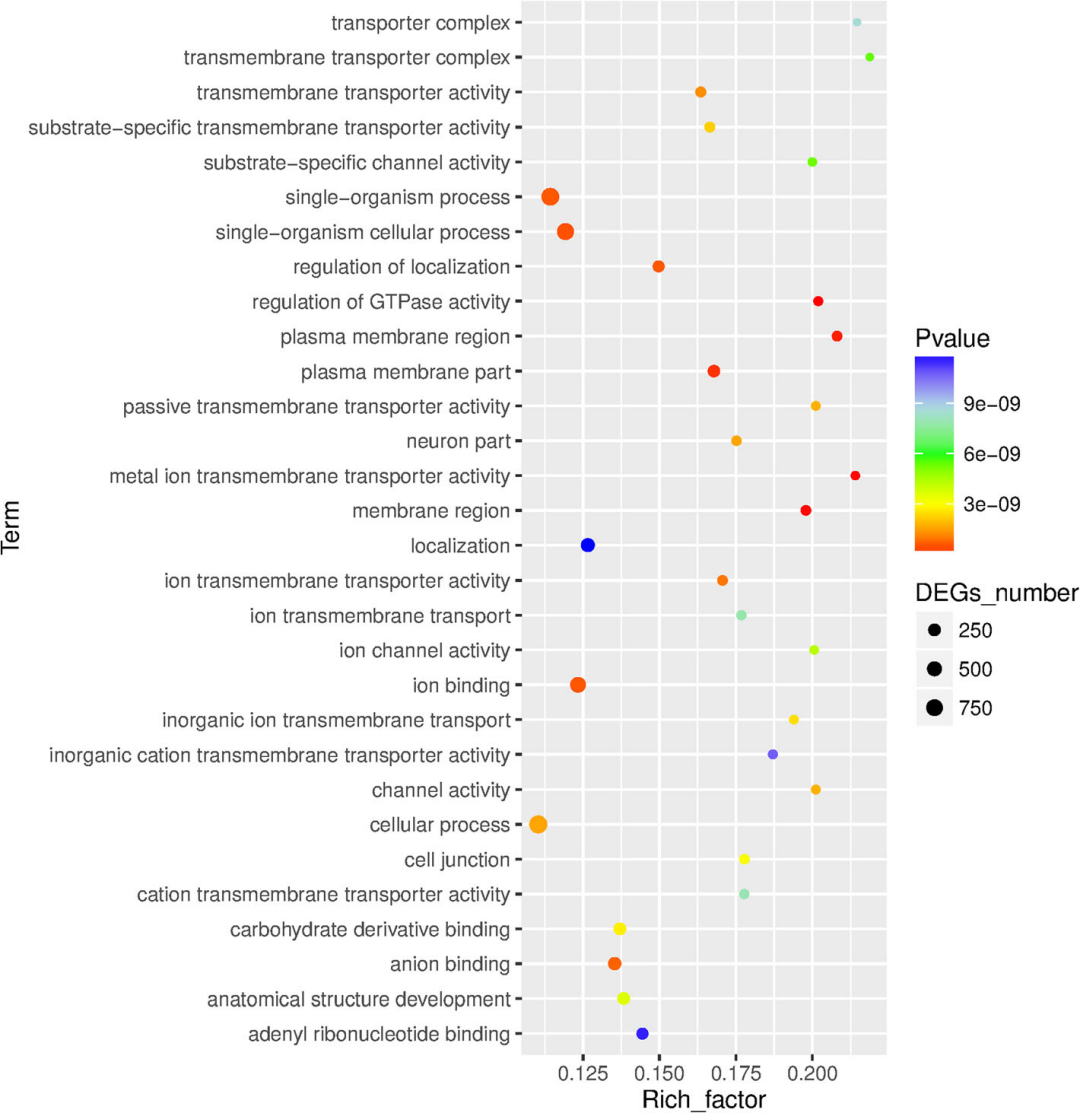
GO enrichment analysis of the ϕC31 integrase target genes. The GO term which satisfied the condition was defined as the GO term which was significantly enriched in the peak-related gene if the Bonferroni corrected *p*-value was < 0.05

**Fig. 5:**
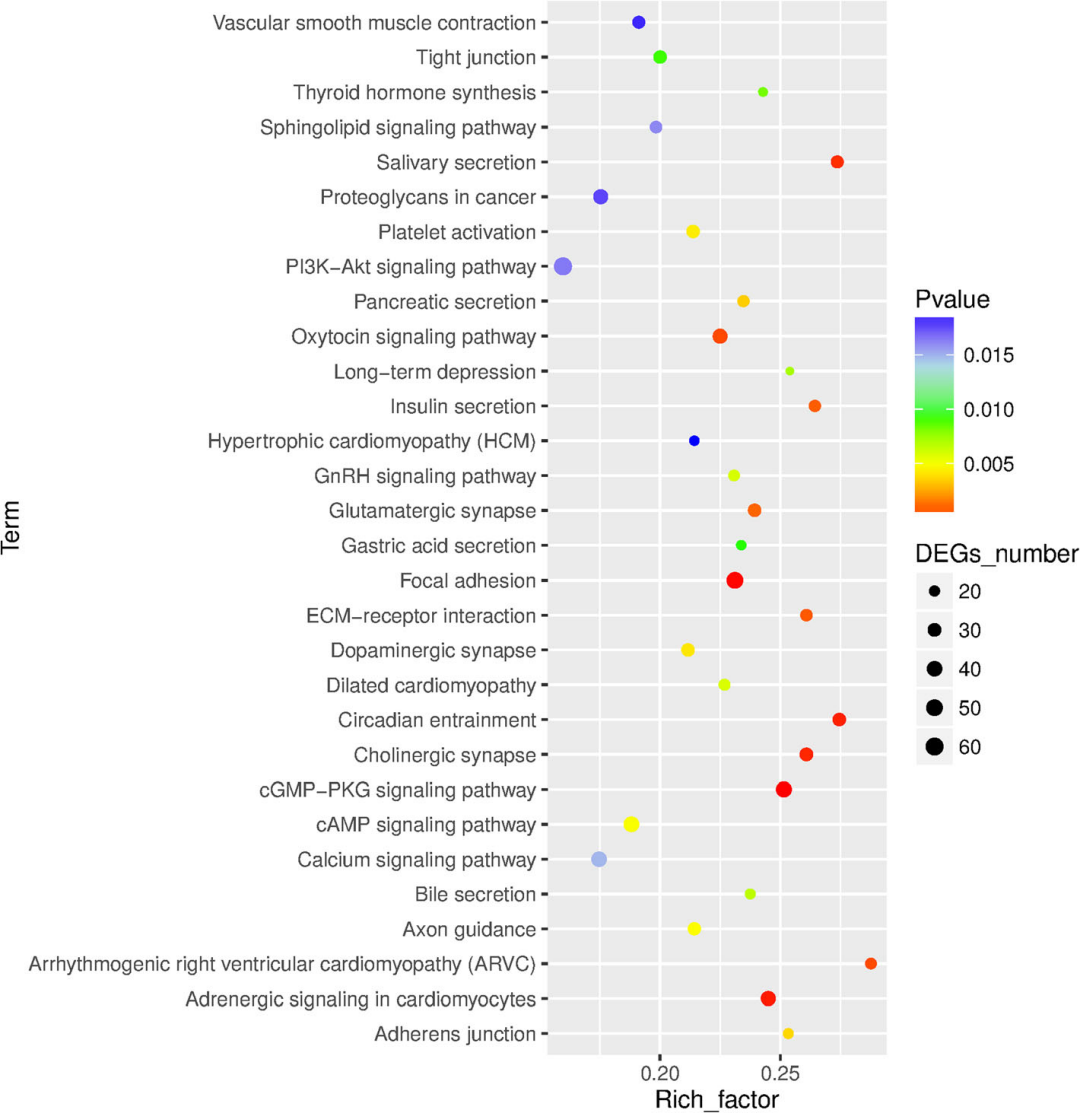
KEGG pathway enrichment analysis of the ϕC31 integrase target genes. KEGG pathway analysis was performed using the KOBAS software The KO term which satisfied the condition was defined as the KO term which was significantly enriched in the peak-related gene if the Bonferroni corrected p-value was < 0.05

To uncover the molecular pathways in which are involved the set of 6740 peaks, we performed GO and KEGG analysesby using bioinformatics tools. In GO functional analysis (Fig. 4, Additional file 1), the most significant GO terms included “regulation of GTPase activity” (GO:0043087; *p-*FDR = 0) for biological process, “metal ion transmembrane transporter activity” (GO:0046873; *p-*FDR = 0) for molecular function, and “membrane region” (GO:0098589; *p-*FDR *= 0*) for cellular component. The Directed Acyclic Graph (DAG) of biological processes, molecular functions and cellular components were subsequently performed in this study to generate the graphical representation of the results of enrichment analysis showing the inclusion relation and the range of functions (Additional file 1: Figures S5-S7). KEGG pathway analysis was performed using the KOBAS software (http://kobas.cbi.pku.edu.cn). The results (Fig. 5, Additional file 5) showed that ΦC31 integrase target genes were significantly associated with “cGMP-PKG signaling pathway” (ko04022, 43 genes,*P* = 2.16E-05), “Focal adhesion” (ko04510,49genes, *P* = 3.95E-05) and “Adrenergic signaling in cardiomyocytes” (ko04261, 37 genes, *P* = 0.000122399).

### qPCR validation

**Fig. 6:**
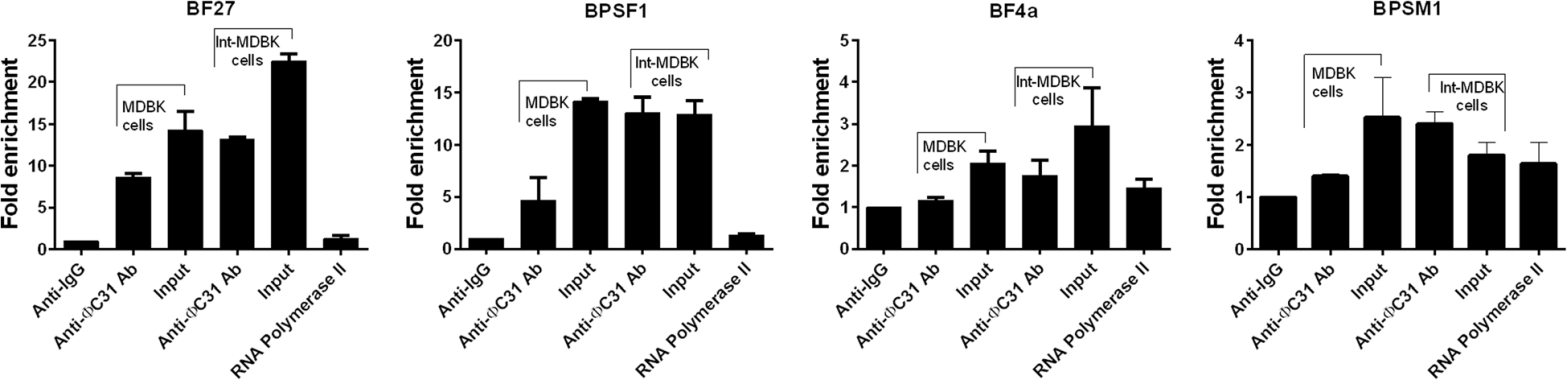
Real time PCR analysis of the ChIP-PCR products of ϕC31 integrase expression for previously studied binding sites

To validate the functional ΦC31 integrase binding to the genome, we performed qRT-PCR assay to verify the binding of ΦC31 integrase to candidate hotspot binding sites, namely BF27, BpsF1, BpsM1 and BF4a that we had found [18]. Indeed, we found that BF27, BpsF1, BpsM1 and BF4a were respectively enriched by 13.2, 13.05, 2.41 and 1.76 fold in the Int-MDKB cells (Fig. 6). These results demonstrated the effective binding of ΦC31 integrase onto target binding sites in the engineered MDBK cells, thus confirming the validity of the ChIP-Seq data at some extent.

## Discussion

The development of genetic engineering techniques for the introduction of gene sequences into mammalian cells has many potential important applications, such as gene therapy, mammary gland bioreactor and mechanism study on gene function [23] The ΦC31 integrase-mediated gene delivery system has the ability to integrate transgenes into mammalian genomes at specific locations, and guarantees high expression levels of recombinant protein [24–33]. Building non-viral expression vectors for ΦC31 integrase will provide an important and safe tool for gene therapy and the generation of transgenic organisms [34–37]. Furthermore, though ΦC31 integrase has been applied for site-directed gene integration in the mammalian genome, the features of ΦC31 integrase binding sites are not well defined. Indeed, although about 10^2^–10^3^ pseudo attP sites have been predicted in mammalian cells [7], the number found in humans and mice is very small. In addition, though we have found 32 integration sites in the bovine genome using reverse nested PCR [18], the actual number of these sites in the genome is very different. Thus, the data on ΦC31 integrase has been found too small to analyze its function and mechanism. Therefore, in this study, we constructed a ΦC31 integrase eukaryotic expression vector and generated a stable MDBK cell line expressing ΦC31 integrase to characterize the binding sites of ΦC31 integrase in the whole bovine genome.

After having confirmed the effective expression of ΦC31 integrase, we applied the ChIP-Seq method to uncover the global map of ΦC31 integrase binding sites. A total of 6740 binding sites were identified to be significantly enriched ΦC31 integrase binding regions in the MDBK cell genome. This study is the first of its kind to uncover the global map of binding sites for ΦC31 integrase in the bovine genome. By analyzing genomic DNA sequences, we identified a highly significant sequence motif among the binding sites. This motif contains inverted repeats and has similarities to wild-type attP site. We also have found that these sites contain repetitive DNA. These results strongly suggest that there are sequence characteristics that define pseudo attP sites in the bovine genome, and that the recognition of these sequences is an important feature of site-specific ΦC31 integrase activity in MDBK cell. Repetitive elements are presumably recognized as a suitable substitute for attP sequences.

Through GO analysis, the most significant GO terms of these genes around the ΦC31 integrase binding sites were “regulation of GTPase activity” in the GO category of biological process, “metal ion transmembrane transporter activity” in the GO category of molecular function, and “membrane region” in the GO category of cellular component. Using KEGG pathway analysis with KOBAS, we found that the genes located in the core significant pathways were associated with “cGMP-PKG signaling pathway” and “Focal adhesion”. These results may explain why the ΦC31 integrase does not necessitate external energy and cofactors as it is able to catalyze the metabolism of energy and microelement pathways. Maybe this hypothesis is wrong because we didn’t have enough direct evidences. Also we don’t know what influence will happen to the location genes expression if a foreign gene is integrated nearby by ΦC31 integrase.The present study has some limitations since we analyzed only a single ChIP-Seq dataset without animal in vivo experimental validation following ChIP-Seq. However, due to the fact that the engineered cells were well characterized and the ChIP-Seq results were validated by real time PCR, it is probable that sequences obtained here are potential ΦC31 integrase target binding sites. This time, our purpose was to validate that the ChIP-seq method can be used to uncover the global binding sites by phiC31 integrase and next time we will take some strategies to optimize our selecting system, in order to get rid of the random binding sites of phiC31 integrase in the bovine genome.

## Conclusion

Our study uncovered a large set of ΦC31 integrase binding sites in the bovine genome and analysis the features of these binding sites. This method will help us to fully understand the mechanism of the site-specific integration function by phiC31 integrase and will potentially boost its genetic manipulations in both gene therapy and generation of transgenic animals.

## Methods

### Plasmid construction

The pCMV-Int plasmid harboring ΦC31 integrase was a kind gift of Prof. M. P. Calos (Stanford University School of Medicine, Stanford, USA). The plasmid pCMV-Int was amplified by PCR using specific forward (5-AAGCTTGGTACCGGTCCGGAATTCCC-3) and reverse (5-CTCGCTGGATCCGGGTGTCGCTAC-3) primers. After digestion by HindIII and xhoI restriction enzymes, the ΦC31 gene fragment was isolated from the PCR product and cloned into the recipient plasmid pcDNA3.1-neo digested by the same restriction enzymes after overnight connection at 16 °C to generate ΦC31 integrase eukaryotic expression vector pcDNA3.1-int-neo.

### Establishment of MDKB cells expressing ΦC31 and verification

MDKB cells were obtained from the Cell Bank of the Chinese Academy of Sciences. All reagents were obtained from Invitrogen unless otherwise indicated. The cells were maintained on dulbecco’s modified eagle medium (DMEM). Fresh medium was provided to the cells every day, and the cells were passaged every 4–5 days. One day prior to transfection with Lipofectamine 2000 (Invitrogen), cells were treated with Accutase (Sigma-Aldrich, St. Louis) and plated on Matrigel in conditioned medium. Lipofectamine 2000 transfection was carried out according to the manufacturer’s protocol. We typically used 4 μg of the ΦC31 integrase expression vector pcDNA3.1-int-neo to transfect 2 million cells. After 14–21 days of selection, individual colonies of MDKB cells transformed with the expression vector were manually picked and expanded for further analysis. DNA was extracted from the transgenic colonies and negative MDKB cells with the phenol chloroform procedure.

The ΦC31 integrase expression was validated by western blot analysis. MDBK cells were washed with 3 ml 4 °C precooled PBS and heated at 80 °C for 30 min in RIPA buffer(150 mM Nacl, 50 mM Tris-HCL pH 8, 0.1% SDS, 0.5% Deoxycholate, 1 mM EDTA, 1% NP-40), and then centrifuged for 5 min at 10000 g. Next, 20 μg total proteins were eledectrophoresed in a 10% SDS-PAGE gel, and then electrically transferred onto PVDF membranes using a semidry method. ß-actin was used as endogenous control. Detection was performed using the ECL-plus enhanced chemiluminescence system (ECL Amersham Biosciences) after auto radiographic exposure to HyperfilmTM.ECL (Amersham Biosciences). The program ImageMaster I-D (Pharmacia, Sweden) was used for quantitation of bands by densitometry.

### Chromatin immunoprecipitation, ChIP-sequencing and ChIP-qPCR

MDBK or Int-MDKB cells (expressing ΦC31 integrase) (1 × 10^7^ cells) were inoculated into 10 mL DMEM medium and cultured to reach 80–90% confluence. Thereafter, cells were fixed with 1% formaldehyde for 15 min cross-linking and quenched with 450 μL 2.5 M glycine. After washing cells three times with ice-cold PBS, chromatin was isolated by adding 1 ml SDS lysis buffer (150 Mm Nacl, 1% Triton X-100, 25 mM Tris pH 7.5, 0.1%SDS, 0.5% Deoxycholate) followed by disruption with a Dounce homogenizer. Lysates were sonicated (VCX750) to shear the DNA to an average length of 200–500 bp, followed by centrifugation at 15000 g for 10 min to remove cell debris. 300 μL of supernatant were divided into 3 aliquots of 100 μL: 100 μL for the experimental group into which was added 1–10 μg anti-ΦC31 antibodies, 100 μL for the control group without addition of antibodies and 100 μL to which was added 4 μL 5 M NaCl. The 3 aliquots were submitted to 3 h cross-linking at 65 °C followed by agarose gel electrophoresis for the evaluation of ultrasonic disruption results. Thereafter, 900 μL ChIP Dilution Buffer (0.01%SDS, 1% Triton X-100, 1% Triton X-100, 1.2 mM EDTA, 16.7 mM Tris-Hcl, 167 mM Nacl pH 8.1) and 20 μL 50 × PIC were added in the experimental and control samples, and cleared with 60 μlProtein AAgarose/Salmon Sperm DNA. 20 μL genomic DNA (input) was purified from both aliquots of chromatin and quantified on a Nanodrop spectrophotometer. Extrapolation to the original chromatin volume allowed quantitation of the total chromatin yield. ChIP assays were carried out as follows: An aliquot of chromatin (50 mg) was precleared with protein Aagarose beads (Invitrogen). ΦC31-bound genomic DNA fragments were isolated using an anti-bacteriophage ΦC31 integrase antibody (ab93248). After incubation at 4 °C overnight, protein Aagarose beads were used to isolate the immune complexes. Complexes were washed, eluted from the beads using SDS buffer (1%SDS, 0.1 M NaHCO_3_), and subjected to RNase and proteinase K treatment. Crosslinks were reversed by incubation overnight at 65 °C, and ChIP DNA was purified by PCR Purification Kit (QIAGEN).

We generated one DNA library using the ΦC31 integrase-specific antibodies incubated with crosslinked and sonicated DNA from Int-MDKB cells. A second control library containing the input DNA was prepared from MDBK cells. About 20–30 ng of the ChIP DNA from each library was prepared for sequencing using the Illumina TruSeq Nano DNA Sample Preparation Kit according to the kit manual. The DNA libraries were sequenced with HiSeq 2500 machine using TruSeq Rapid SBS Kit-HS reagents (Illumina).

To ensure the accuracy of subsequent bioinformatics analysis, the original sequencing data was filtered to obtain high-quality sequencing data (clean data). Quality control of the sequencing data was performed using Sickle (https://github.com/najoshi/sickle) and SeqPrep (https://github.com/jstjohn/SeqPrep). The sequencing output raw reads were trimmed by stripping the adaptor sequences and ambiguous nucleotides and reads with quality scores less than 20 and lengths below 20 bp were removed.

Equal amounts of DNA for each sample were combined with a SYBR Green PCR master mix (Qiagen) and specific primers. Amplification reactions were then performed with an Applied Biosystems 7900 real-time PCR system. The primers used for BF27 binding site were 5-GTACTTATTTTTGTATTTGAAACCACCT and TCTGGGTGTTTTTACATT-3; for BF4a, 5-CCATAAAAGGAATATACTTGAAA-3 and 5-ACCAACATGGCAATCGGGGACAT-3; for BPSF1, 5-GGACCTAGAAGGGGCTCATAACT-3 and 5-TGTATACTCACACCACTGTCCTA-3; for BPSM1, 5-GCCGTTTGTTGGGTTAGCTTTTCAGAA-3 and 5-GTCATCCTGCCTTGCAGGCTGTTTCTT-3; for GAPDH, 5-TTCACCACCATGGAGAAGG-3 and 5-GGCATGGACTGTGGTCATGA-3. Use AppliedBiosystems SDS2.4 and RQ manager software to analyze data.

### ChIP-Seq library peak finding

To identify significant ΦC31 binding sites in the sequencing data, the whole genome peak scan was performed using the UCSC Genome Browser following the UCSC Genome Browser Instructions (http://genome.ucsc.edu). UCSC provides genomic reference sequences for a large number of species, including a large number of gene annotation information and comparative genomics information.

### MEME motif discovery analyses

The MEME web service (http://meme-suite.org) was ran with the command line summary below: meme INTMDBK_treat.peaks.fa -dna -mod zoops -revcomp -nmotifs3 -maxw30 -minw 6 -maxsize 26,000,000 -oc. /INTMDBK_treat_meme.GO functional enrichment analysis of Peak related genes.

Gene Ontology (GO) is an internationally standardized gene function classification system that provides a set of dynamically updated standard vocabularies to comprehensively describe the properties of genes and gene products in organisms. GO has a total of three ontologies describing the molecular function of the gene, the cellular component in which it is located, and the biological process involved. GO is the basic unit (term, node), each term corresponds to a property.

The GO enrichment analysis was used to map all the peak-related genes to terms in the Gene Ontology database (http://www.geneontology.org/) and calculate the number of genes for each term. The hypergeometric test was applied to find out significant GO enrichment of peak-related genes based on the entire genome background using the formula [38]:

$$ P=1-\sum \limits_{i=0}^{m-1}\frac{\left(\begin{array}{c}M\\ {}i\end{array}\right)\left(\begin{array}{c}N-M\\ {}n-i\end{array}\right)}{\left(\begin{array}{c}N\\ {}n\end{array}\right)} $$

Where N is the number of genes with GO annotation in the genome; n is the number of peak-related genes in N; M is the number of genes annotated for a particular GO term in the genome; m is the number of peak-associated genes annotated for a particular GO term. The GO term which satisfied the condition was defined as the GO term which was significantly enriched in the peak-related gene if the Bonferroni corrected *p*-value was < 0.05. The Directed Acyclic Graph (DAG), a graphical representation of the results of enrichment analysis of peak-related genes was also equally performed.

### KEGG pathway enrichment analysis

This study used KOBAS (http://kobas.cbi.pku.edu.cn) for KEGG pathway enrichment analysis. The calculation principle was the same as GO enrichment analysis. Terms with corrected p-value of < 0.05 was considered significant.

## Additional files

Additional file 1: GO functional analysis output file. **Figure S1.** Genome sequencing depth cumulative distribution obtained from the INPUT sample. **Figure S2.** Gene and upstream and downstream sequences depth distribution map obtained from the INPUT sample. **Figure S3.** Genome sequencing depth cumulative distribution obtained from the INTMDBK_treat sample. **Figure S4.** Gene and upstream and downstream sequences depth distribution mapobtained from the INTMDBK_treat sample. **Figure S5.** Directed Acyclic Graph (DAG) of “Biological Processes” obtained from GO enrichment analysis. **Figure S6.** Directed Acyclic Graph (DAG) of “Cellular Components” obtained from GO enrichment analysis. **Figure S7.** Directed Acyclic Graph (DAG) of “Molecular Functions” obtained from GO enrichment analysis. (ZIP 726 kb)

Additional file 2: Captured ϕC31 integrase binding sites (markers) information and location. (XLSX 15 kb)

Additional file 3: The peak calling output file and peak annotation are reported. (XLSX 109 kb)

Additional file 4: KEGG functional analysis output file. (XLSX 855 kb)

Additional file 5: GO functional analysis output file. (XLSX 29 kb)

## Abbreviations

Chip-seq: Chromatin immunoprecipitation-DNA sequencing
GO: Gene Ontology
KEGG: Kyoto Encyclopedia of Genes and Genomes
MDBK: Maden-darby bovine kidney
MEME: Multiple Em for Motif Elicitation
ΦC31: PhiC31
