# Supplementary figures and images for: Global mapping of binding sites for phic31 integrase in transgenic maden-darby bovine kidney cells using ChIP-seq
Source: Hereditas. 2019 Jan 14;156:3. doi: 10.1186/s41065-018-0079-z (PMC6332687; doi:10.1186/s41065-018-0079-z)

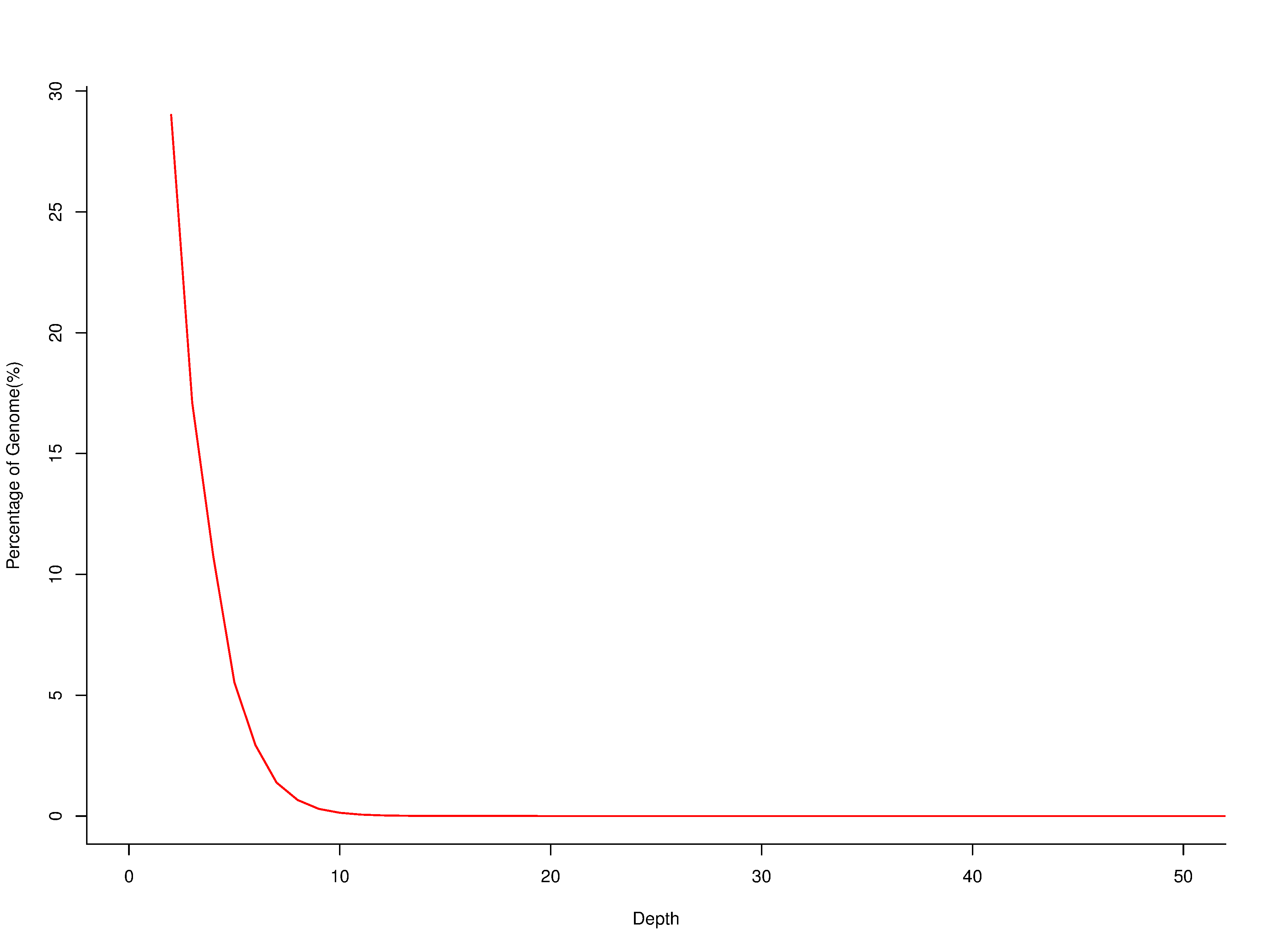

Supplement: Supplementary file 1 — GO functional analysis output file. Figure S1. Genome sequencing depth cumulative distribution obtained from the INPUT sample. Figure S2. Gene and upstream and downstream sequences depth distribution map obtained from the INPUT sample. Figure S3. Genome sequencing depth cumulative distribution obtained from the INTMDBK_treat sample. Figure S4. Gene and upstream and downstream sequences depth distribution mapobtained from the INTMDBK_treat sample. Figure S5. Directed Acyclic Graph (DAG) of “Biological Processes” obtained from GO enrichment analysis. Figure S6. Directed Acyclic Graph (DAG) of “Cellular Components” obtained from GO enrichment analysis. Figure S7. Directed Acyclic Graph (DAG) of “Molecular Functions” obtained from GO enrichment analysis. (ZIP 726 kb) [file 41065_2018_79_MOESM1_ESM.zip › Figure S1.tiff]

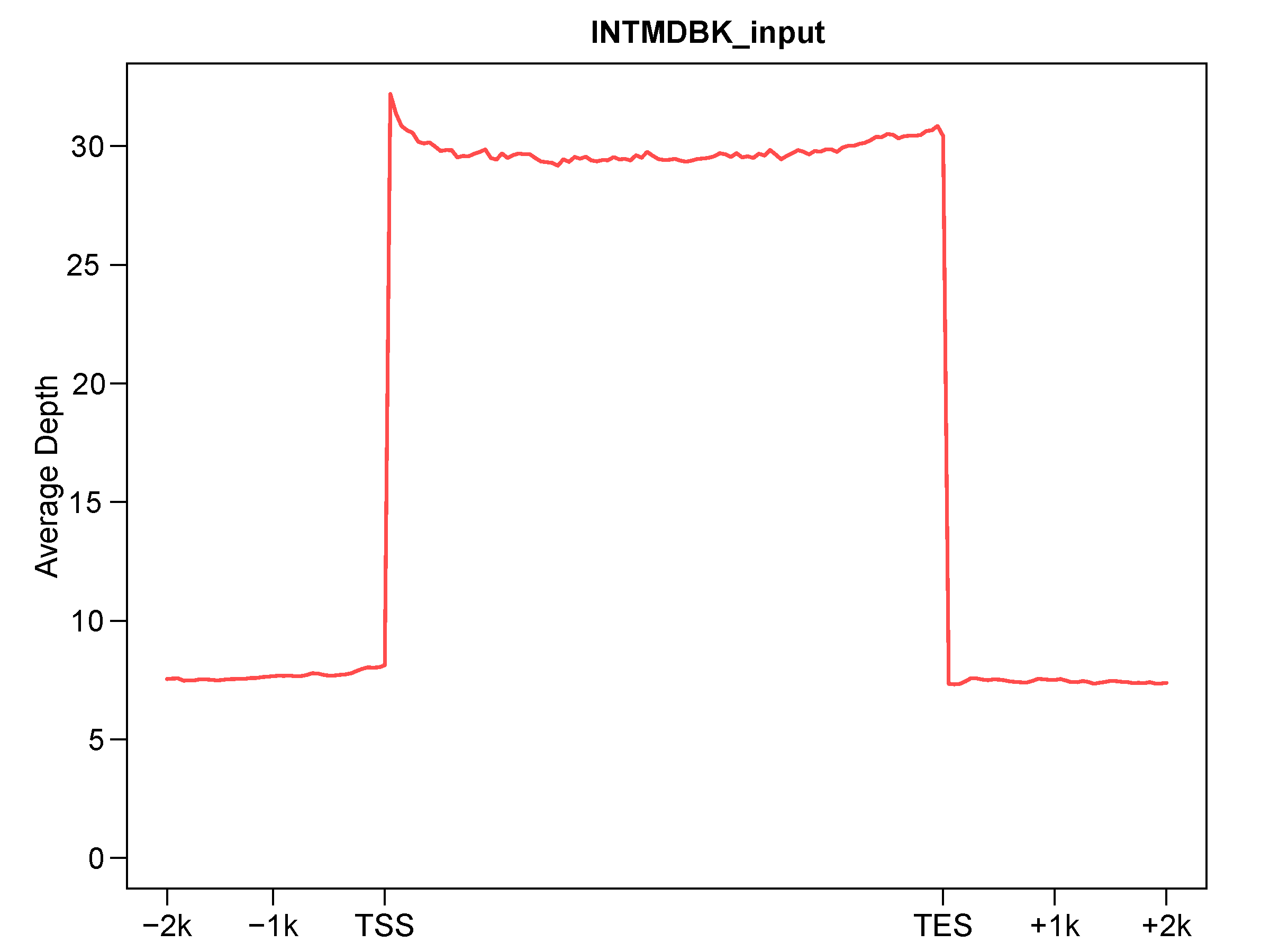

Supplement: Supplementary file 1 — GO functional analysis output file. Figure S1. Genome sequencing depth cumulative distribution obtained from the INPUT sample. Figure S2. Gene and upstream and downstream sequences depth distribution map obtained from the INPUT sample. Figure S3. Genome sequencing depth cumulative distribution obtained from the INTMDBK_treat sample. Figure S4. Gene and upstream and downstream sequences depth distribution mapobtained from the INTMDBK_treat sample. Figure S5. Directed Acyclic Graph (DAG) of “Biological Processes” obtained from GO enrichment analysis. Figure S6. Directed Acyclic Graph (DAG) of “Cellular Components” obtained from GO enrichment analysis. Figure S7. Directed Acyclic Graph (DAG) of “Molecular Functions” obtained from GO enrichment analysis. (ZIP 726 kb) [file 41065_2018_79_MOESM1_ESM.zip › Figure S2.tiff]

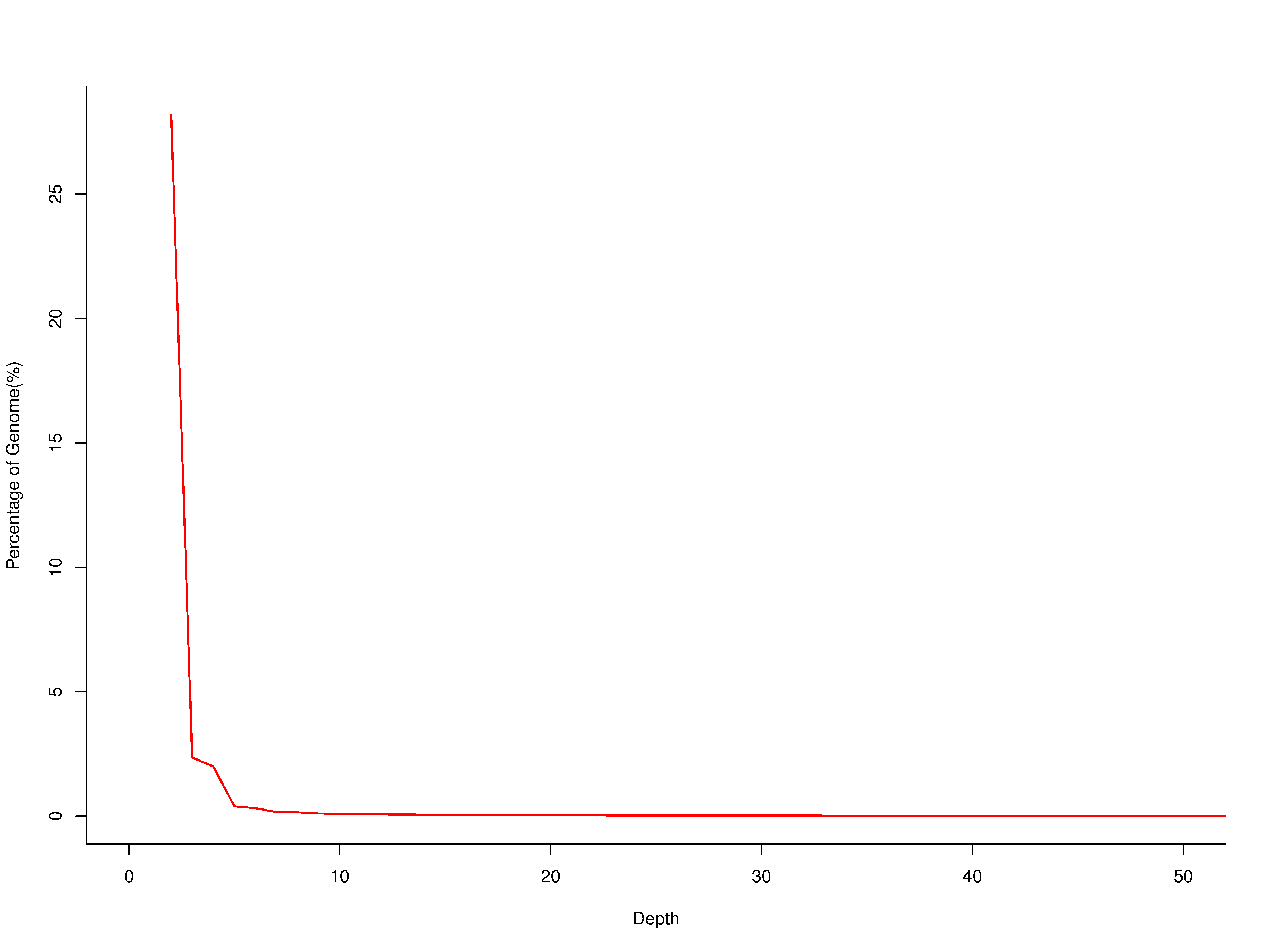

Supplement: Supplementary file 1 — GO functional analysis output file. Figure S1. Genome sequencing depth cumulative distribution obtained from the INPUT sample. Figure S2. Gene and upstream and downstream sequences depth distribution map obtained from the INPUT sample. Figure S3. Genome sequencing depth cumulative distribution obtained from the INTMDBK_treat sample. Figure S4. Gene and upstream and downstream sequences depth distribution mapobtained from the INTMDBK_treat sample. Figure S5. Directed Acyclic Graph (DAG) of “Biological Processes” obtained from GO enrichment analysis. Figure S6. Directed Acyclic Graph (DAG) of “Cellular Components” obtained from GO enrichment analysis. Figure S7. Directed Acyclic Graph (DAG) of “Molecular Functions” obtained from GO enrichment analysis. (ZIP 726 kb) [file 41065_2018_79_MOESM1_ESM.zip › Figure S3.tiff]

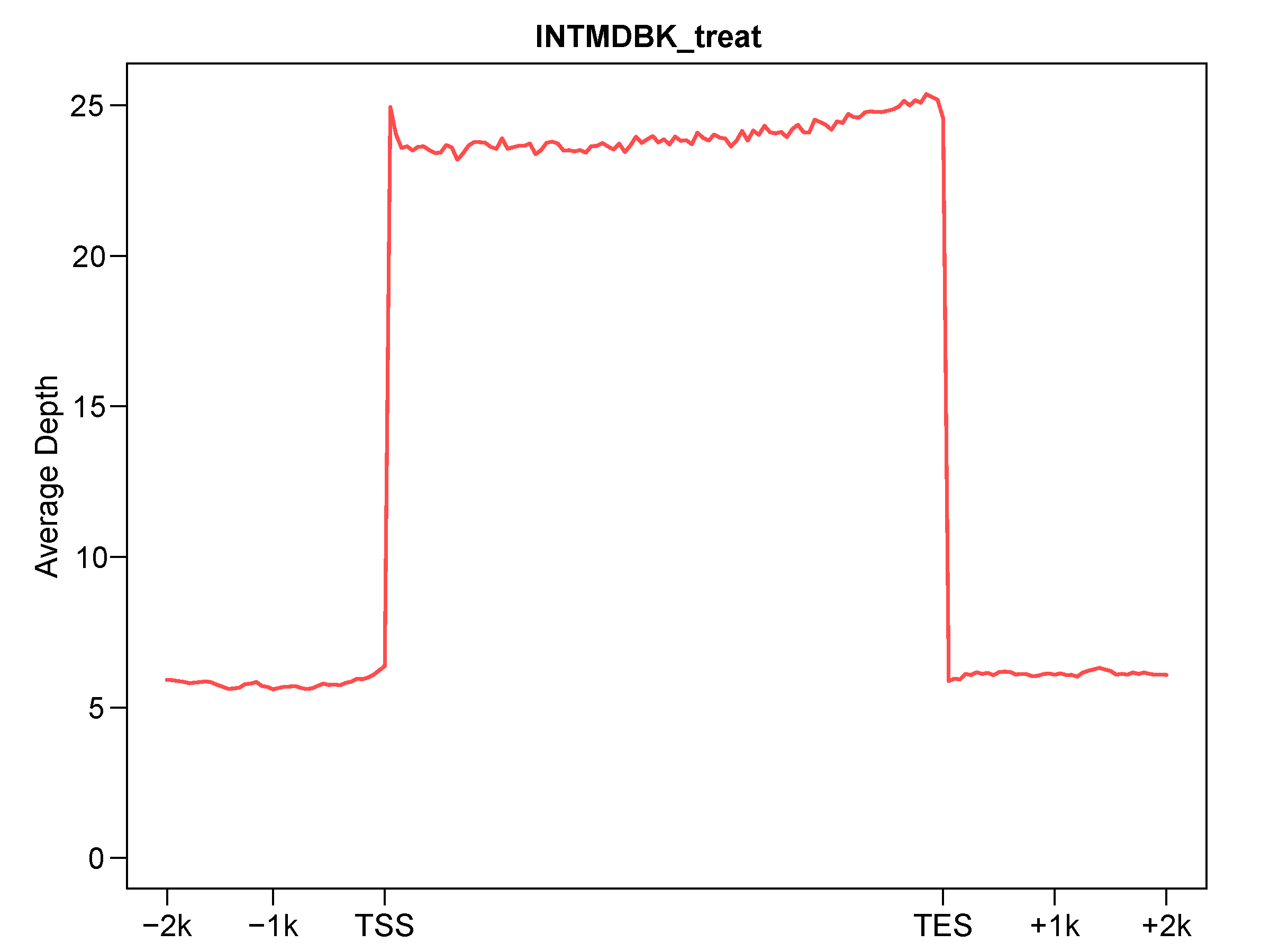

Supplement: Supplementary file 1 — GO functional analysis output file. Figure S1. Genome sequencing depth cumulative distribution obtained from the INPUT sample. Figure S2. Gene and upstream and downstream sequences depth distribution map obtained from the INPUT sample. Figure S3. Genome sequencing depth cumulative distribution obtained from the INTMDBK_treat sample. Figure S4. Gene and upstream and downstream sequences depth distribution mapobtained from the INTMDBK_treat sample. Figure S5. Directed Acyclic Graph (DAG) of “Biological Processes” obtained from GO enrichment analysis. Figure S6. Directed Acyclic Graph (DAG) of “Cellular Components” obtained from GO enrichment analysis. Figure S7. Directed Acyclic Graph (DAG) of “Molecular Functions” obtained from GO enrichment analysis. (ZIP 726 kb) [file 41065_2018_79_MOESM1_ESM.zip › Figure S4.tiff]

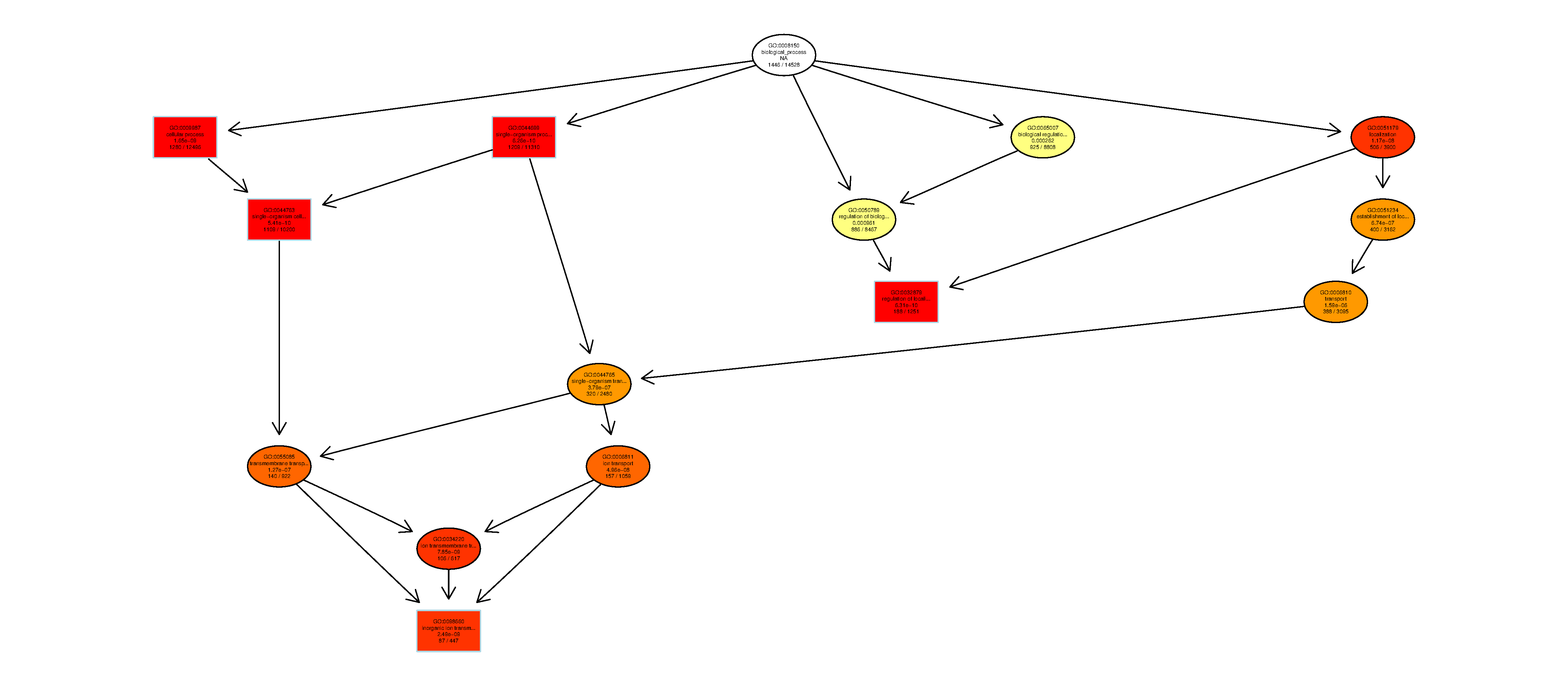

Supplement: Supplementary file 1 — GO functional analysis output file. Figure S1. Genome sequencing depth cumulative distribution obtained from the INPUT sample. Figure S2. Gene and upstream and downstream sequences depth distribution map obtained from the INPUT sample. Figure S3. Genome sequencing depth cumulative distribution obtained from the INTMDBK_treat sample. Figure S4. Gene and upstream and downstream sequences depth distribution mapobtained from the INTMDBK_treat sample. Figure S5. Directed Acyclic Graph (DAG) of “Biological Processes” obtained from GO enrichment analysis. Figure S6. Directed Acyclic Graph (DAG) of “Cellular Components” obtained from GO enrichment analysis. Figure S7. Directed Acyclic Graph (DAG) of “Molecular Functions” obtained from GO enrichment analysis. (ZIP 726 kb) [file 41065_2018_79_MOESM1_ESM.zip › Figure S5.tiff]

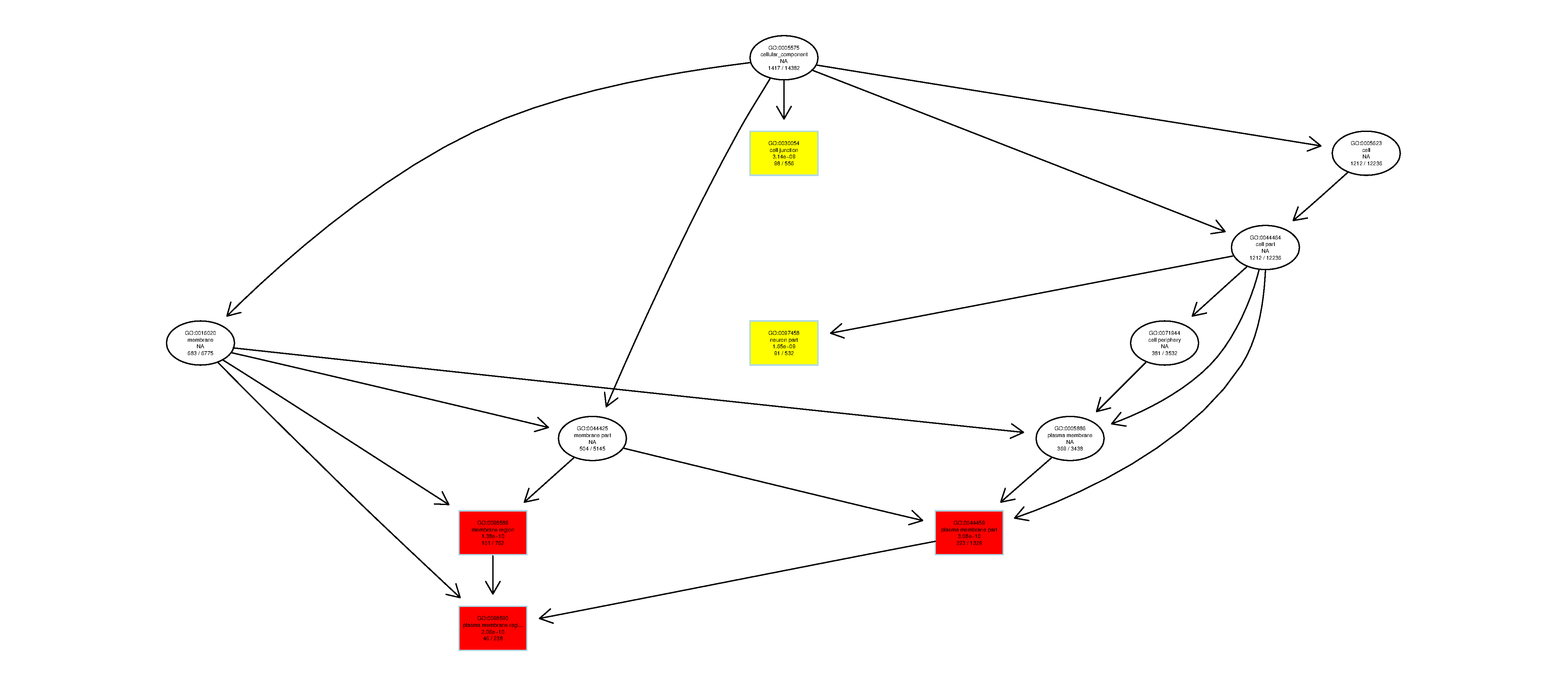

Supplement: Supplementary file 1 — GO functional analysis output file. Figure S1. Genome sequencing depth cumulative distribution obtained from the INPUT sample. Figure S2. Gene and upstream and downstream sequences depth distribution map obtained from the INPUT sample. Figure S3. Genome sequencing depth cumulative distribution obtained from the INTMDBK_treat sample. Figure S4. Gene and upstream and downstream sequences depth distribution mapobtained from the INTMDBK_treat sample. Figure S5. Directed Acyclic Graph (DAG) of “Biological Processes” obtained from GO enrichment analysis. Figure S6. Directed Acyclic Graph (DAG) of “Cellular Components” obtained from GO enrichment analysis. Figure S7. Directed Acyclic Graph (DAG) of “Molecular Functions” obtained from GO enrichment analysis. (ZIP 726 kb) [file 41065_2018_79_MOESM1_ESM.zip › Figure S6.tiff]

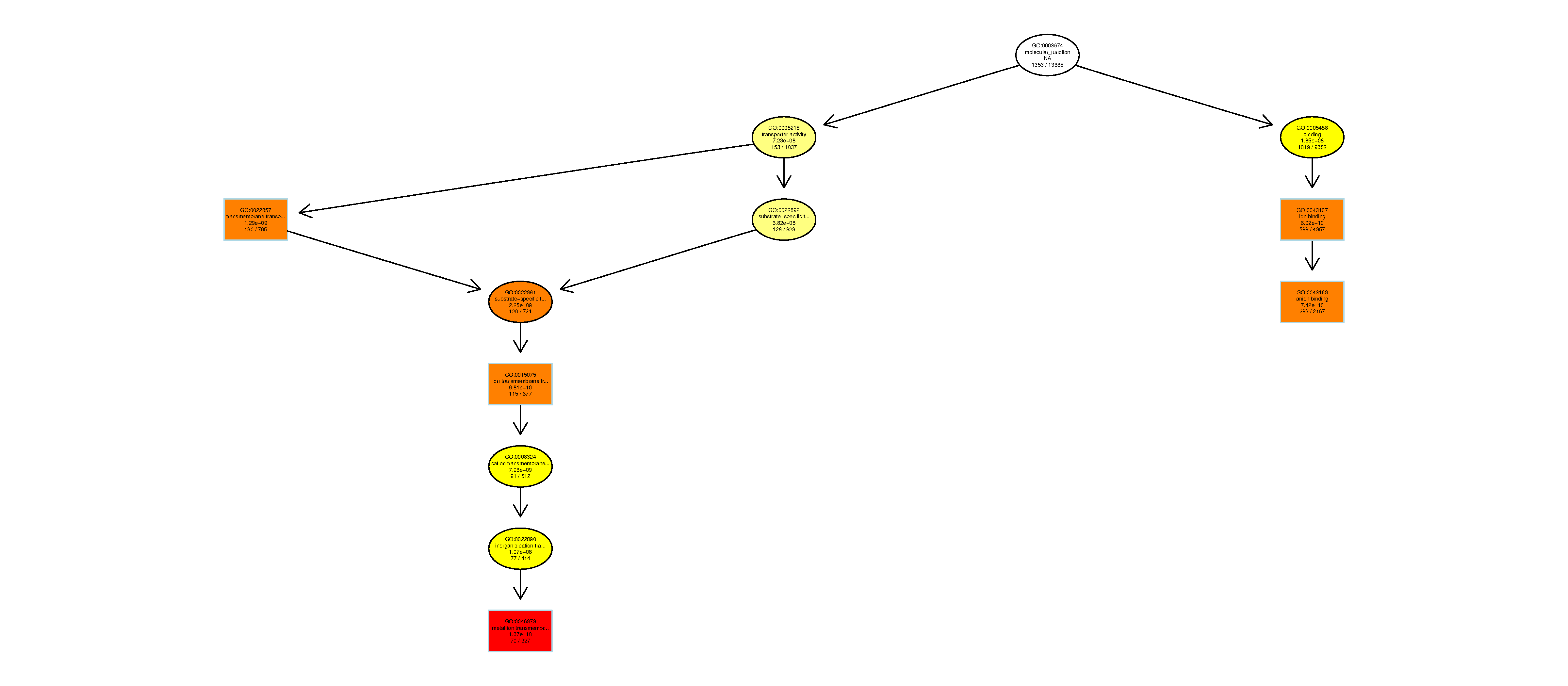

Supplement: Supplementary file 1 — GO functional analysis output file. Figure S1. Genome sequencing depth cumulative distribution obtained from the INPUT sample. Figure S2. Gene and upstream and downstream sequences depth distribution map obtained from the INPUT sample. Figure S3. Genome sequencing depth cumulative distribution obtained from the INTMDBK_treat sample. Figure S4. Gene and upstream and downstream sequences depth distribution mapobtained from the INTMDBK_treat sample. Figure S5. Directed Acyclic Graph (DAG) of “Biological Processes” obtained from GO enrichment analysis. Figure S6. Directed Acyclic Graph (DAG) of “Cellular Components” obtained from GO enrichment analysis. Figure S7. Directed Acyclic Graph (DAG) of “Molecular Functions” obtained from GO enrichment analysis. (ZIP 726 kb) [file 41065_2018_79_MOESM1_ESM.zip › Figure S7.tiff]
